# New Drugs and Preparations

**Published:** 1894-03-17

**Authors:** 


### New Drugs and Preparations.

[We shall be glad to receive, at our Office, 428, Strand, London, W.C., from the manufacturers, specimens of all new preparations and drugs which may be brought out from time to time.]

### SERUM THERAPY.

Arthur Klein[1] points out that the study of blood serum may affect the whole future of medicine. Immunity, which is the power of an organ to defend itself successfully against the entrance of infection, is either congenital and peculiar to the species, or acquired. Two explanations are suggested for acquired immunity. One supposes that the white blood corpuscles take up and destroy the cause of infection. The other supposes that the means of protection consists in substances dissolved in the blood. There are two varieties of excitants of infection. One growing in the tissues, and passing into the circulation, threatens the organism by the enormous number of parasitic micro-organisms. Examples of this are septicæmia and anthrax. In the other the micro-organisms, *e.g.*, those of tetanus, diphtheria, cholera, do not enter the circulation, but produce an intensely active poison which threatens the life of the host. These poisons are called toxines, and any substance which is an antidote to them is an antitoxine. It is believed that the serum of immune animals contains these antitoxines.

Buchner[2] leaves the chemical constitution of toxines doubtful. Broadly speaking, they are toxalbumins, and are the direct products of bacterial cells. With regard to antitoxines, it has been supposed that they and the toxine neutralise each other, but several experiments show that this is not so, either in the test tube or in the body; the antitoxine acts by rendering the animal immune. Buchner concludes by making the very suggestive remark that antitoxines are also bacterial products.

André[3] details two cases of small-pox treated with hypodermic injections of serum obtained from the blood of patients who had previously suffered from the disease. The treatment produced no effect favourable or unfavourable on the evolution of the disorder.

Schneidemühl[4] gives an account of recent researches on the serum treatment for glanders, the bacillus of which is B. mallei. Mallein can be obtained from cultivating the bacillus, and injections of this render possible the diagnosis of doubtful cases. The author gives an account of the work of Boschetti, who finds that the injection with blood serum of horses affected with glanders causes a rise of temperature in animals suffering from the disease, but this is less than that produced by mallein. Hell, he says, has used the serum for curative and protective purposes. In a regiment none of sixty-six horses who received protective injections were affected. Three horses already affected were treated with the serum, and the results were favourable. Hell gives other cases, and concludes that in early cases the disease may be brought to a standstill in three days. Toepper also gives an account of an epidemic of glanders treated by serum, and his results are as favourable as Hell's.

Chernel[5] gives an account of experiments conducted with the blood serum of animals immune against cholera, and, on the whole, he concluded, from the work upon dogs, that it would be possible to render human beings immune against cholera by serum injections, which have no danger in themselves. These experiments were conducted by Pawlowsky and Buchstab.[6]

Hammerschlag[7] gives an account of the serum treatment for typhoid fever. He first refers to Stern's experiments in regard to the immunity conferred upon some animals by the blood of patients suffering from enteric fever. Also he describes how five patients suffering from this fever were transfused with blood from those convalescent from it. In the first three patients no effect was produced; in the fourth there was a striking fall of temperature, but it was very temporary; in the fifth the patient died, quite independently of the treatment. No certain conclusion can, therefore, be drawn from these cases. The author refers to the apparent negative results obtained by Chantemesse and Vidal,[8] who found that, while the injections did no harm, they apparently did no good, but they only operated upon two patients.

[1] Therapeutische Blätter, No. 1, 1893. [2] Münchener Medicinische Wochenschrift, 1893, Nos. 24 and 25. [3] Archives Cliniques de Bordeaux, July, 1893. [4] Deutsche Medicinische Wochenschrift, Feb. 16, 1893. [5] Wiener Medicinische Blätter, No. 24, 1893. [6] Deutsche Medicinische Wochenschrift, No. 22, 1893. [7] Deutsche Medicinische Wochenschrift, July 27, 1893. [8] Les Nouveaux Remèdes, Feb., 1893.

### DIURETIN.

Massalongo and Silvestri[1] have made very extensive researches on diuretin, or, as it should be more correctly called, theobromine sodio-salicylate. They have used it in all sorts of cases in which a diuretic was desirable, and they conclude that (1) diuretin is a pure diuretic, and acts by stimulation of the renal cells and renal tissue, increasing the flow of urine even in those cases in which the heart muscle is too feeble to respond to treatment. (2) The diuretic action is most marked in cardiac or renal dropsy. The drug is of little use in ascites due to cirrhosis, or in serous effusions. (3) The diuretic acid is continued after the drug has been discontinued. (4) In ordinary doses diuretin does not give rise to unpleasant consequences, neither has it any cumulative action. (5) In the earlier stages of heart disease it is not nearly so useful as the digitalis group of drugs, but when œdema appears it is most beneficial. It acts best in mitral disease. (6) With regard to kidney disease, good results are most evident in acute nephritis. (7) Sometimes the diuretic action is delayed for several hours after the drug has been given. Herrick[2] has also tried this drug, He, like the last authors, thinks it most useful for heart cases withd ropsy. He also agrees that it is not of much use in cirrhosis of the liver, nor in chronic intestinal nephritis. He, however, finds that occasionally it produces nausea, vomiting, diarrhœa, palpitation, and a rash on the skin. The maximum daily amount that can be given with safety is 150 grains, but usually between 50 and 120 grains suffice. It should be given in milk without acids, and between meals. Genersich[3] tried this drug on sixteen patients. He also finds that it is most useful for cardiac dropsy, and not of much value for cirrhosis or serous effusions. He believes that it will be of great value, and considers that the only disadvantages are its price and the unpleasant symptoms, which, like the last author, he sometimes met with. He generally gave 60 to 90 grains a day. Cohnstein[4] has studied experimentally the action of this drug. He finds that it has no effect on the blood pressure, no constant influence on the pulse, and no effect on the energy of the cardiac contractions. Therefore, presumably, its good effects are due to its influence on the renal cells. These results refer to ordinary doses. If poisonous doses are given, the animal dies from profound cardiac collapse. Pawinski[5] also agrees that diuretin does not act directly on the heart to any great extent, but he thinks that the slight rise of blood pressure which he sometimes obtained was due to a slight cardiac and vaso-motor stimulation. Like all other authors he regards it as in the main acting on the renal cells. He has occasionally observed the disagreeable effects already mentioned. He considers it a very valuable drug for cases of heart disease with œdema, and for cases of acute nephritis. He usually gives 45 to 70 grains a day, and thinks it acts best when given in solution.

[1] Rif. Med., No. 58, 1893. [2] Journal of the American Medical Association, Mar. 11, 1893. [3] Centralblat f. Therap., March, 1893. [4] Berl. Klin. Wochenschrift, No. 4, 1893. [5] Vratch, 1893, No. 13.

### GALLOBROMOL.

Lepine[1] has brought this drug under the notice of the profession. It is the pharmaceutical name for dibromogallic acid, which is gallic acid in which two atoms of hydrogen are replaced by two of bromine. It contains about half its weight of bromine, while potassium bromide contains two-thirds, hence larger doses are required of it than of potassium bromide. It consists of white, very delicate needles, very soluble in alcohol, ether, and boiling water, and sufficiently soluble in cold water for ordinary prescribing. The only disagreeable effects produced by it are occasional heaviness in the gastric region and occasional gastric pain. It certainly soothes those patients who are usually benefited by bromide of potassium, and is possibly preferable, as it is so much less depressing. The dose has to be determined by experiment in every case, but the desired effect is usually obtained with from thirty to fifty grains in twenty-four hours. As it has somewhat an acid taste, it is usually desirable to conceal this with some pleasant tasting syrup.

[1] The Medical Week, 1893, Vol. I., p. 338, and Vol. II., p. 535.

### NEW PREPARATION OF BISMUTH.

On adding a solution of a bismuth salt to an alkaline phenate a dirty white precipitate is obtained, consisting of phenol-bismuth, metacresol-bismuth, and naphthol-bismuth. Researches undertaken by Jasenski[1] shows that in the stomach and small intestines these substances are split up into phenol, cresol, or naphthol and bismuth. All the phenol and cresol are eliminated by the kidneys in combination with sulphur or glycuronic acid, a portion only of the naphthol is so excreted, the bismuth is entirely eliminated by the intestine. Experiment showed that 60 grains in twenty-four hours of either of these substances could be given for weeks without any ill-results, and they appear likely to be of great value for the form of diarrhœa for which bismuth is commonly employed.

[1] Arch. des Sciences Biologiques de St. Petersbourg, T. ii., No. 2, 1893.

